# Supplementary material for: Update on Nutritional Advice Post-Heart Transplant: A Cross-Sectional Study across French-Speaking European Centers
Source: Nutrients. 2024 Aug 25;16(17):2843. doi: 10.3390/nu16172843 (PMC11397603; doi:10.3390/nu16172843)

Supplementary data:

Supplementary Table S1: Identified heart transplant centers in the French-speaking European area.

City	Hospital	Number of heart transplantation
**France*[1]**		
Bordeaux	CHU Bordeaux	36
Caen	CHU Caen Normandie	4
Clermont-Ferrand	CHU de Clermont-Ferrand	4
Créteil	Hôpital Henri Mondor	5
Dijon	CHU Dijon	10
Grenoble	CHU Grenoble Alpes	10
Le Plessis-Robinson	Hôpital Marie Lannelongue	11
Lille	CHRU de Lille	36
Lyon	Hôpital Louis Pradel	31
Marseille	Hôpital La Timone APHM	21
Montpellier	CHU de Montpellier	21
Nancy	CHRU de Nancy	13
Nantes	CHU de Nantes	16
Paris	Hôpital Bichat Claude-Bernard	37
	Hôpital Universitaire La Pitié Salpêtrière	90
	Hôpital Européen Georges-Pompidou	21
Rennes	Hôpital Pontchaillou	17
Rouen	CHU Rouen Normandie	4
Strasbourg	Les Hôpitaux Universitaires de Strasbourg	11
Toulouse	CHU de Toulouse	19
Tours	Hôpital Trousseau	10
**Belgium**		
Bruxelles	Hôpital Erasme	15*[2]
	Cliniques universitaires Saint-Luc	13**[3]

Liège	CHU de Liège	12***[4]
**Switzerland**		
Lausanne	Centre hospitalier universitaire vaudois	20****[5]
Genève	Hôpitaux Universitaires de Genève	§

*CHU : University Hospital, APHM : Assistance Public des Hôpitaux de Marseille, CHRU : Regional University Hospital. ; *data of 2019 ; **data of 2022 ; ***data of 2015 ;* ****data of 2020 *;* § Geneva patients are operated on in Lausanne but followed up in Geneva. The number of transplants is included in those performed in Lausanne ;

[1]*Komajda M, Gandjbakhch I, Dorent R, Leprince P, Trochu JN, Welty C. Rapport 20-05–La transplantation cardiaque chez l'adulte. Bulletin de l'Académie Nationale de Médecine. 2021;205(2):111–117. ;*

[2]*Creteur J. Le Service des Soins Intensifs de l'Hôpital Erasme (Cliniques Universitaires de Bruxelles). Anesthésie & Réanimation. 2020;6(1):50–53. ;*

[3]*https://www.saintluc.be/fr/faits-chiffres ;*

[4]*Bruls S, Tchana-Sato V, Lavigne JP, Durieux R, Sakalihasan N, Radermecker M, et al. La transplantation cardiaque : indications actuelles et resultats de l'experience liegeoise. Revue Médicale de Liège. 2020;75(1).* [5]*https://rapportsannuels.chuv.ch/*

Supplementary Table S2: Number of centers per advice.

Criteria	Number of centers n(%)
**Environmental hygiene and food preparation: duration of advice**	
No environmental hygiene and food preparation recommended	2 (8%)
Environmental hygiene and food preparation for 2 months	1 (4%)
Environmental hygiene and food preparation for 3 months	1 (4%)
Environmental hygiene and food preparation for 6 months	4 (15%)
Environmental hygiene and Food Preparation for 1 year	2 (8%)
Environmental hygiene and food preparation for life	1 (4%)
Environmental hygiene and food preparation without duration	15 (58%)
**Food eviction: Duration of recommandation**	
No food eviction recommended	2 (8%)
Food eviction for 2 months	1 (4%)
Food eviction for 3 months	1 (4%)
Food eviction for 6 months	6 (23%)
Food eviction for 1 year	6 (23%)
Food eviction without specified duration	10 (38%)
Restaurant / Outside meal for 3 months	1 (4%)
Restaurant / outside meal for 1 year	1 (4%)
**Nutritional advice**	
Varied/balanced diet	18 (69%)
Regular physical activity	7 (27%)
Enriched food to fight against malnutrition	3 (12%)
Digestive disorders diarrhea / constipation	1 (4%)
Avoid alcohol (no end date)	6 (23%)
Avoid alcohol for 3 months	1 (4%)
**Protein advice**	
Sufficient protein intake	4 (15%)
OPV at 2 meals	2 (8%)
OPV 1 to 2	5 (19%)
**Advice Lipids**	
Control lipids	14 (54%)
Limit saturated fats and fatty products (pastries...)	7 (27%)
Enrich with oleaginous fruits	1 (4%)
**Advice Carbohydrates**	
Control carbohydrate intake	2 (8%)
Regulate complex carbohydrates	8 (31%)

Limit simple carbohydrate intake	20 (77%)
Limit fruit	12 (46%)
**Fiber advice**	
Fiber intake	4 (15%)
**Sodium advice**	
Controlled salt intake 2-3g	1 (4%)
Controlled Salt Intake 4g	1 (4%)
Controlled Salt Intake 5-6g	2 (8%)
Controlled Salt Intake 6-8g	1 (4%)
Customized Controlled Salt Intake	6 (23%)
Controlled salt intake (unspecified)	8 (31%)
Limit sodium-rich water	10 (38%)
Individual water restriction	1 (4%)
**Calcium advice**	
Adequate calcium intake (unspecified)	1 (4%)
Adequate calcium intake 1 to 1.2g/day	3 (12%)
3/5 dairy products / day	5 (19%)
Water rich in calcium	4 (15%)
2 dairy products/day	2 (8%)
**Daily hygiene advice**	
Good hand hygiene (soapy water)	21 (81%)
Wash kitchen environment (unspecified)	6 (23%)
Wash kitchen environment with soapy water	7 (27%)
Wash kitchen environment with vinegar water	4 (15%)
Wash kitchen environment with bleach water	13 (50%)
Use dishwasher only	1 (4%)
Change the sponge regularly	2 (8%)
Clean cloth of the day	2 (8%)
Avoid contact with cats and excrement	2 (8%)
Garden with gloves	2 (8%)
Avoid animals in the kitchen	2 (8%)
Avoid contact with pet reptiles	1 (4%)
**Food hygiene**	
**General**	
Respect the cold chain	20 (77%)
Respect the expiration dates	20 (77%)
Avoid cooking in the microwave	1 (4%)
Clean fruit and vegetables (not specified)	10 (38%)
Clean fruit and vegetables rinsed with water	5 (19%)
Clean fruit and vegetables with vinegar water	9 (35%)
Clean fruit and vegetables in bleach water	2 (8%)

Product without veterinary estempille	2 (8%)
Home-made products / take-away	10 (38%)
Avoid cut-up food	15 (58%)
Avoid home frozen products	1 (4%)
Avoid home canned food	2 (8%)
Avoid canned or brick products	1 (4%)
Avoid multi-portion packaging	10 38%)
Avoid unpackaged food	8 (31%)
Bulk food	8 (31%)
Avoiding leftovers	3 (12%)
Avoiding leftovers beyond 24 hours	6 (23%)
Avoid leftovers beyond 48 hours	6 (23%)
Put leftovers in the refrigerator within 2 hours	6 (23%)
Store cooked and raw products separately	7 (27%)

Specific foods evictions	
Raw meat, fish, eggs, cold cuts	24 (92%)
Smoked products	15 (58%)
Horse meat	2 (8%)
Offal	3 (12%)
Raw, unsterilized or pasteurized cheese/milk/dairy	23 (88%)
Rind of cheeses	11 (42%)
All cheeses	1 (4%)
Raw cheese allowed if cooked	3 (12%)
Raw shellfish and mollusks	15 (58%)
Raw and cooked shellfish	5 (19%)
Sprouted seeds	7 (27%)
Dried fruits and olaginous	1 (4%)
Raw vegetables outside the home	1 (4%)
Raw vegetables (fruit and vegetables)	2 (8%)
Fresh fruit juice	1 (4%)
Spices and herbs added after cooking	8 (31%)
Commercial pastries with uncooked risk food (cream, egg)	5 (19%)
Tap water, ice cubes, filtering carafe	6 (23%)

Prohibited in case of immunosuppressive drugs	
Grapefruit	24 (92%)
Pomelo	6 (23%)
Tangelo	3 (12%)
Chinese grapefruit	2 (8%)
Bitter orange	10 (38%)
St. John's wort	18 (69%)
Carambola	5 (19%)
Pomegranate	6 (23%)
Lime	1 (4%)
Bergamot	1 (4%)

Licorice	1 (4%)
Ginseng Root	1 (4%)
Citron	2 (8%)

Supplementary Figure S1: Number of centers per sodium intake recommendation (in grams of salt) (n=26).

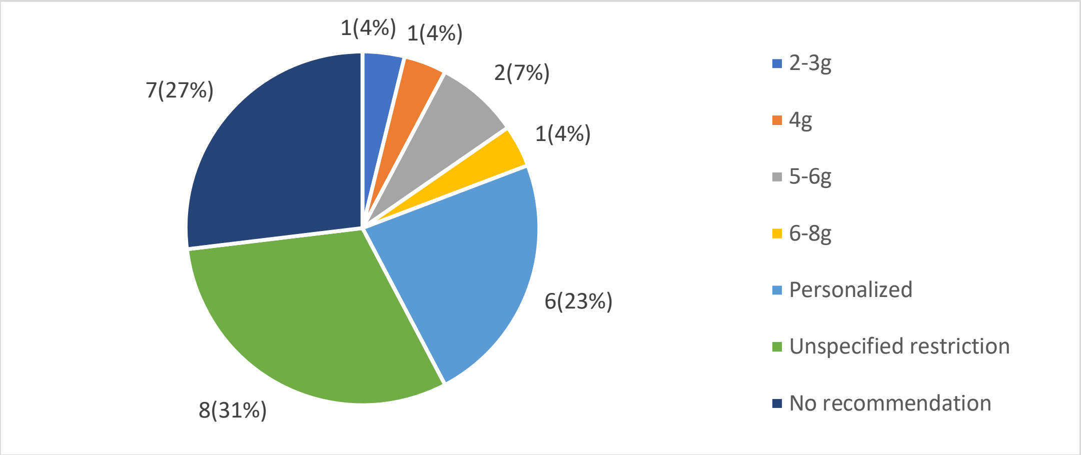

Supplement: Supplementary file 1 [file nutrients-16-02843-s001.zip › nutrients-3139732-supplementary.pdf]
